# Dental genomics in Africa: colonial legacies and research gaps

**DOI:** 10.3389/froh.2025.1655867

**Published:** 2025-11-20

**Authors:** Salma Kabbashi, Imaan A. Roomaney, Manogari Chetty

**Affiliations:** Department of Craniofacial Biology, Pathology, & Radiology, Faculty of Dentistry, University of the Western Cape, Cape Town, South Africa

**Keywords:** African, colonial legacies, dental genetics, genomics, research

## Abstract

Oral health disparities are closely linked to broader health inequalities, particularly in global health contexts where disproportionate emphasis is placed on diseases other than oral health. In the field of dental genetics, recent investigations have highlighted persistent challenges and barriers in African genomic research. Colonial legacies continue to influence the structuring of research agendas and contribute to the marginalization of indigenous knowledge systems. We discuss the implications of these historical dynamics for the relevance of genetic research findings, and addresses the emerging ethical considerations in clinical applications and community engagement. We emphasize the need for equitable and culturally inclusive approaches to expand our genetic understanding of dental pathologies in underrepresented African populations.

## Introduction

Oral health is a critical yet chronically neglected component of global health, particularly in low- and middle-income countries (LMICs). According to the 2022 *WHO Global Oral Health Status Report*, oral diseases affect 3.5 billion people- more than any other non-communicable disease (NCD). An estimated 45% of the world's population is affected by one or more untreated oral disease (1). Increasing evidence links oral health to systemic conditions such as cardiovascular disease, metabolic disorders, neurological diseases, and adverse maternal outcomes (2–5). Despite these associations, oral health remains marginal in health policy and funding agendas, especially in sub-Saharan Africa (1, 6).

In this region, common NCDs like dental caries and periodontal disease pose significant public health challenges, exacerbated by limited access to care and underdeveloped research infrastructure (1, 7). Compounding this neglect is the global underinvestment in oral health genomics. Advances in dental genomics offer opportunities to uncover population-specific risk factors and advance precision dentistry, yet most genomic studies have centred on Euro-American populations. This imbalance raises serious concerns about equity, scientific validity, and the applicability of findings across global populations (8). To further contextualize this gap, we conducted a focused literature search across databases, which confirmed that Africa-based genomic studies in oral health remain sparse, with only a few verified primary outputs in the most recent years.

### Colonial legacy and eurocentric biases in oral health research

Historical and structural determinants rooted in colonialism continue to exert a significant influence on oral health research and care across the African continent. During the colonial era, health systems, including dental services, were primarily established to serve the needs of colonial administrators and urban elites, with minimal investment in the health infrastructure of rural or indigenous populations (9). Western biomedical models were imposed upon local populations, frequently at the expense of indigenous knowledge systems, which were often dismissed as unscientific or irrelevant. These colonial patterns have left a lasting imprint on contemporary research agendas, particularly in the field of human genetics.

A significant indicator of this legacy is the pronounced under-representation of African populations in global genomics research. Foundational initiatives, such as the Human Genome Project and the International HapMap Project, as well as early genome-wide association studies (GWAS), largely excluded African participants. According to Popejoy and Fullerton (2016), by 2016, over 80% of GWAS participants were of European ancestry, with individuals of African descent comprising less than 4% (10). By 2021, African representation in GWAS studies had decreased to just 1.1%, reflecting a rapidly growing genomics data gap (11). More recent analyses confirm that this disparity is accelerating despite increasing calls for equity in genomic research (8, 12). As a result, genetic risk loci for complex diseases, including those affecting oral health, have been disproportionately identified and validated in Euro-American cohorts. This skewed representation has significant implications; risk alleles identified in European populations may not be informative, or may even be misleading, when applied to African cohorts due to differences in allele frequencies, linkage disequilibrium structures, and gene-environment interactions (13). As a result, the clinical translation of genomic findings into diagnostics and therapeutics is severely constrained in African settings.

For instance, polymorphisms in the interleukin-1 (IL-1) gene cluster were once considered predictive markers for the severity of periodontitis in European populations (14–18). However, their utility in African-ancestry individuals remains contested, due to population-specific allele frequencies, gene-gene interactions, and the broader lack of contextual validation (19). In fact, significant resources have gone into development tests for IL-1 polymorphisms as diagnostic tools for periodontal disease, with little validation on diverse populations (20–24). This challenge is further compounded by the scarcity of studies across the continent, largely attributed to limited resources, infrastructure, and investment in genomic research. As evidence increasingly links periodontal disease to systemic conditions, it is clear that periodontal disease cannot be viewed in isolation as merely an “oral” disease (2, 3, 5).

Although Africa bears one of the world's largest burdens of oral disease, genomic investigation into common oral conditions has remained marginal, a pattern that reflects colonial-era research priorities. While initiatives like H3Africa have sequenced thousands of African genomes and identified millions of novel variants, these projects have not focused on common oral diseases. This omission perpetuates knowledge gaps and limits the potential for precision oral healthcare tailored to African populations. In oral health research specifically, the dearth of genomic data from African populations limits the development of predictive models and biomarker discovery relevant to prevalent diseases such as periodontitis and dental caries. Furthermore, existing bioinformatic tools and reference panels are often optimised for non-African genomes, leading to biases in variant calling and interpretation (25).

The legacy of colonialism is also evident in the neglect of oral health in both policy and research domains. Despite the high burden of oral diseases in Africa, oral health has received scant attention from major funding bodies and governments (7). Several authors highlight the paradox that Africa possesses the greatest human genetic diversity globally, yet remains underexplored in studies of the genetic basis of oral diseases (7, 26, 27). Oral diseases are frequently framed as the result of individual behaviours, such as poor dietary habits, inadequate hygiene, or lifestyle choices, rather than being recognised as complex, multifactorial NCDs (6, 28). This oversimplified view overlooks the significant roles of genetic susceptibility, host-microbiome interactions, and broader structural determinants, including healthcare access and socioeconomic conditions (28, 29). A critical step toward addressing oral health inequities is acknowledging that individuals affected by these conditions are not solely responsible for their development. The concept of “preventability” in diseases such as dental caries and periodontal disease is far more complex than simply promoting access to toothbrushes and toothpaste; it requires a deeper understanding of the social, biological, and structural factors that influence health outcomes.

### Underrepresentation of African populations and the emerging field of African dental genomics

Despite the resource challenges, a growing body of work has sought to characterise the genetic architecture of oral health conditions in African populations. Recent studies from initiatives such as H3Africa have begun to lay the groundwork for African genomics in general, although investigations specific to dental health remain sparse (30). Research on craniofacial anomalies, tooth agenesis, and susceptibility to caries is slowly emerging, often led by multidisciplinary teams that include oral health clinician-scientists, geneticists, and epidemiologists (7).

One notable area of focus is the exploration of inflammatory markers and immune-regulatory genes such as IL-1, TNF-α, and MMPs in relation to periodontal disease (27, 29). However, findings are often inconsistent across populations, highlighting the necessity of large-scale, locally led studies that consider environmental, microbial, and behavioural covariates. Additionally, the intersection of genomics with developmental disorders and syndromic presentations of oral disease presents new avenues for translational research and targeted interventions in African contexts. Particularly in the microbial-genetic intersection, referred to as infectogenomics, there is growing evidence of population-specific bacterial and genetic profiles in diseases that are highly prevalent but least investigated in Africa, such as periodontitis (27).

In oral health research specifically, the lack of African genomic data hinders the development of predictive models and biomarker discovery for diseases such as periodontitis and dental caries. Existing bioinformatic tools and reference panels are often optimized for non-African genomes, leading to biases in variant calling and interpretation (25). Sudi et al., (2022) emphasize that although Africa carries a high burden of oral disease, genomic studies in this field remain extremely limited. While initiatives like H3Africa have sequenced thousands of African genomes and identified millions of novel variants, these projects have not focused on common oral diseases. This omission perpetuates knowledge gaps and limits the potential for clinical translation and precision oral healthcare tailored to African populations. Furthermore, the exclusion of both oral diseases and African participants from large-scale health studies leads to fragmented research efforts, unnecessary duplication, and inefficient use of resources (29).

To substantiate this gap, we conducted a focused literature search (2021–2025) across PubMed, Science Direct, Scopus, and Google Scholar. Eligible studies were restricted to those involving African participants and addressing genetic or genomic aspects of oral or craniofacial conditions. Both primary research and contextual/review papers were considered, whereas diaspora-only cohorts, non-dental genomics, and laboratory-only studies and singular case reports were excluded. Out of 255 screened records, 16 studies met the inclusion criteria, comprising 10 primary genomic investigations and six reviews or context papers. Table 1 presents the primary Africa-based genomic studies, while Table 2 presents reviews and contextual analyses that underscore the broader structural gaps. Taken together, the evidence map demonstrates that Africa-based genomic outputs in oral health are still modest relative to disease burden. No large-scale GWAS of caries or periodontitis have yet been conducted on African cohorts. Reports of rare disorders, such as *FAM20A-*related Enamel Renal Syndrome, remain at the level of case series, and replication studies are underpowered. Reviews and bibliometric analyses confirm that capacity remains uneven across countries, with research concentrated in a few centres.

**Table 1:** Africa-based primary genomics studies in oral/dental health (2019–2025).

Author	Phenotype/Condition	Country/Population	Study design & genomic method	Sample	Key verified finding
Olatosi et al. (31)	Early childhood caries (ECC)	Nigeria/Children <6 years old	Candidate variant replication of GWAS loci; genetic risk assessment	Pilot Study	Replicated selected GWAS caries loci in a Nigerian children with ECC cohort; demonstrates feasibility but highlighted the need for adequately powered African GWAS.
Chetty et al. (32)	Osteogenesis imperfecta (OI), Pyle disease and Torg-Winchester syndrome	South Africa	Genotype-phenotype correlation study	66 individuals with skeletal dysplasias	12 individuals had tauradontism
Gowans et al. (33)	Orofacial clefts with co-occurring clubfoot	Ghanaian probands	Whole-exome sequencing (Agilent SureSelect XT + Illumina HiSeq2500), with Sanger validation)	Families/cohort (study level)	WES implicated multiple syndromic loci in African families with co-occurring phenotypes, highlighting genetic heterogeneity and pleiotropy in African cohorts. Deep phenotyping is needed.
Awotoye et al. (34)	Non-syndromic orofacial clefts	Ethiopia, Ghana, and Nigeria	genome-wide gene × sex (GxSex) interaction study	1,019 non-syndromic orofacial cleft cases and 2,159 controls	Identification of a new risk locus associated with OFC in an African population and an 8p22 locus whose association with nsOFC is driven by GxSex interaction.
Gowans et al. (35)	Non-syndromic cleft lip and/or cleft palate	Ghana, Ethiopia, and Nigeria	parent-of-origin effects from GWAS data	174 case-parent trios	Possible parent of origin effects identified.
Naiker et al. (36)	Van der Woude/*IRF6*-related orofacial clefting (OFC)	South Africa (Durban)	Targeted gene panel/Sanger confirmation focused on *IRF6*	Case series/cohort (clinical genetics)	Identified pathogenic/likely pathogenic *IRF6* variants in South African patients with OFCs (e.g., VDW/Popliteal pterygium), highlighting utility of targeted testing and local counselling pathways
Oladayo et al. (37)	Non-syndromic orofacial clefts	Ghana and Nigeria	WGS at 30x coverage	130 case-parent trios	Identification of 246 unique variants in 55 genes. Five variants in four genes were classified as pathogenic or likely pathogenic
Roomaney et al. (38)	Enamel Renal Syndrome (ERS; *FAM20A*)	Sub-Saharan Africa (multi-site)	Clinical genetics with molecular confirmation (*FAM20A*)	4 unrelated patients	Confirmed *FAM20A* pathogenic variants in African patients with ERS; defines oral/craniofacial profile and emphasizes need for rare-disease genomics capacity in Africa.
Alade et al. (39)	Cleft lip and/or palate	Nigeria, Ghana, and Ethiopia	Gene-based analysis of previously published GWAS	814 non-syndromic cleft lip with or without palate (NSCL/P), 205 non-syndromic cleft palate only (NSCPO), and 2,150 unrelated control	Five genes (ABCB1, TTC28, PDZD8, CENPF, ALKBH8) were previously associated with NSOFCs or diseases presenting with cleft phenotypes. The remaining three genes (CSAD, EXPH5, SLC16A9) are potentially novel candidate genes for NSOFCs.
Kallala et al. (40)	Dental Fluorosis	Tunisia	Candidate gene association study	95 participants including 51 cases and 44 controls	Significant association of COL1A2 (rs 412,777) polymorphism in the COL1A2 gene with dental fluorosis.

**Table 2:** Selected recent reviews and contextual studies relevant to African dental genomics (2019–2025).

Author	Scope	Relevance to African dental genomics	Key verified points
Sudi et al. (7)	Genetics of oral diseases in Africa (mini-review)	Directly addresses African under-representation	Documents very limited African genomic data across caries, periodontitis, oral cancer, Noma; calls for GWAS, gene–environment, sequencing, and capacity-building.
Olujitan et al. (41)	Periodontal diseases in Africa (review)	Epidemiology/burden; genetics referenced	High prevalence/severity; genetics mentioned but little African primary genomic output, illustrates the evidence gap for periodontitis genomics in Africa.
Kabbashi et al. (29)	Omics→ dental practice (LMIC lens)	Systems/translation, including Africa	Outlines translational barriers (infrastructure, skills, funding) and the need to link omics to dental care in LMICs, supporting your capacity arguments.
Sandhu et al. (42)	Allele-frequency disparities in caries risk variants	Global comparison including African ancestry data	Shows population frequency differences for caries-associated alleles; highlights that many loci were discovered outside Africa and lack African replication.
Kanmodi et al. (43)	Nigeria orofacial cleft research (bibliometric)	Output mapping	Confirms modest OFC research throughput; supports your claim that genetic/OFC outputs remain limited and uneven within Africa.
Rotimi et al. (44)	Cancer genomics in Africa (umbrella)	Methodological/structural parallels	Highlights overall paucity of African cancer genomics, transferable to oral oncology contexts (e.g., OSCC), reinforcing systemic under-representation.

### Indigenous knowledge systems and traditional oral health practices

The neglect of oral health is further compounded by the marginalisation of traditional African oral hygiene practices, which are historically dismissed rather than empirically evaluated for potential health benefits (45).

Traditional oral hygiene practices, including the use of chewing sticks (e.g., *Salvadora persica*) and herbal remedies, have long been employed across African societies. These practices offer culturally embedded strategies for oral health maintenance and have demonstrated antimicrobial and anti-inflammatory properties in laboratory settings (45). Yet, their exclusion from formal biomedical discourse has roots in colonial epistemologies that privileged Western scientific paradigms over indigenous systems of knowledge.

Recent scholarship has called for a critical re-evaluation of traditional oral health practices using rigorous scientific frameworks. Ethnopharmacological studies and community-based participatory research approaches are particularly valuable for assessing the efficacy, safety, and mechanisms of these practices, which can potentially inform the development of accessible and culturally appropriate oral health interventions (46, 47). Research by Agbor and Naidoo (2015, 2016) has demonstrated the significant role traditional healers play in the prevention and management of oral diseases in African contexts (48, 49). Their studies also highlight a substantial gap in the integration of traditional medicine into formal healthcare systems, as well as the need for systematic evaluation of treatment outcomes related to traditional dental care. Similar findings have emerged from studies conducted in South Africa and Nigeria, which emphasize the untapped potential of ethnomedicinal approaches and the need for further investigation (50, 51).

Despite these promising insights, there appears to be limited progression into the experimental and laboratory-based evaluation of traditional medicinal herbs. This lack of follow-through may stem from insufficient investment, institutional barriers, or the perceived lack of prestige associated with research on traditional medicine. Understanding and addressing these barriers is essential to fully explore and validate the role of indigenous knowledge in advancing oral health equity.

### Research capacity and ethics in African genomics

Historically, genomic research in Africa has been characterized by external leadership, limited local capacity, and extractive practices, a model often critiqued as “helicopter science” (52). This dynamic not only undermines sustainable research ecosystems but also raises ethical concerns about data sovereignty, community engagement, and equitable benefit sharing.

Programs such as H3Africa and the African Society of Human Genetics have made substantial progress in reversing these trends by fostering African-led research networks, training early-career scientists, and establishing biorepositories and bioinformatics hubs across the continent (53). Furthermore, the H3Africa initiative sought to develop appropriate ethical regulatory frameworks to govern research in these areas (54). Ethical frameworks tailored to African contexts are increasingly being considered to guide responsible research conduct, including protocols for informed consent, data governance, and the return of results (55). In addition, ethical principles rooted in African philosophies- such as Ubuntu, which emphasizes interconnectedness, communal dignity, and mutual responsibility- have gained prominence as guiding frameworks for responsible genomic research (56).These values challenge the individualistic paradigm dominant in Western bioethics. These principles offer a strong foundation for ethical decision-making in genomic research. Ubuntu promotes ethical frameworks that focus on relationships, responsibilities, and the long-term benefit of the community (56). Unlike traditional models that focus mainly on individual autonomy, this approach recognises the roles of families, community leaders, and traditional authorities in guiding participation in research- a significant shift from the traditional approaches (57).

At the same time, there is growing recognition of the risks of applying universalized ethical standards without contextual adaptation- Africa is in no way homogenous. The challenge lies in aligning global principles, such as autonomy, data access, and benefit sharing, with culturally relevant practices that do not hinder innovation or delay urgently needed health benefits for African communities (58). The H3Africa Data and Biospecimen Access Committee (DBAC) has implemented safeguards to ensure that data access reflects principles of equity and reciprocity, including requirements for collaboration with African scientists and the return of benefits to African populations. However, a recent report noted a surprisingly low number of data access requests from African institutions, likely reflecting ongoing challenges in local capacity, including a lack of bioinformatics expertise and computational infrastructure. This represents a deeply concerning barrier, especially given the continent's unique genomic diversity and public health needs (59).

These ethical and structural considerations are not unique to dental genomics. However, they are directly relevant to oral health and dental genomics, which remain underrepresented in both research and policy agendas. The 2024 *WHO Guidance for Human Genome Data Collection, Access, Use and Sharing,* emphasizes equity, inclusion, and contextual relevance in genomic research (60). Yet, oral health continues to be overlooked, despite being a significant contributor to the burden of disease and disability in many African countries. Addressing this gap requires a reconfiguration of how health priorities are defined and who is included in those decisions.

### Reclaiming representation, ethics, and innovation

To dismantle colonial legacies and address the persistent underrepresentation of African populations in genomics, particularly in dental genomics; a truly inclusive research agenda must centre African voices, ethics, and priorities. This requires a shift toward community-driven research models that are culturally grounded and responsive to local needs. It also entails the deliberate integration of traditional knowledge systems, expansion of genomic sampling to include underrepresented rural and linguistic communities, and investment in local bioinformatics and AI-driven technologies.

In this context, Foláyan et al. (2025) called for a rights-based, accountability-informed decolonisation framework in global oral health- one that actively redistributes power and places respect for Indigenous and marginalised communities at its core (61). They argue that oral health must be recognised as a human right, and that governments and health systems bear the responsibility to address historical and structural inequities. This approach demands culturally relevant care, inclusive policymaking, and meaningful community participation; not merely as beneficiaries, but as co-creators of knowledge and agents of their own health (61, 62). Such a framework fosters solidarity, prioritises Indigenous leadership, and urges the oral health community to “learn to unlearn” entrenched Euro-American ideologies in favour of more equitable and justice-oriented systems (61).

Finally, pan-African collaboration platforms are essential to counter the fragmentation imposed by colonial-era boundaries, while public education campaigns and investments in Afrocentric biotechnology can foster trust and restore local ownership of scientific agendas. Gender equity must also be foregrounded, along with the inclusion of dental genomics in national health strategies, to ensure that oral health research and services reflect the diversity, values, and realities of African populations. Ultimately, reclaiming representation and innovation in African genomics requires structural transformation- not just in what research is done, but in how, by whom, and for whose benefit.

## Conclusion

The current landscape of dental genetics research in Africa is deeply influenced by colonial legacies. 46 of the 47 countries in the WHO Africa Region grapple with the consequences of colonisation. African populations remain underrepresented in genomic datasets, while Eurocentric paradigms continue to shape research questions and methodologies. Additionally, indigenous knowledge systems have been systematically sidelined. While colonial legacies continue to shape knowledge production and research infrastructure; emerging initiatives demonstrate the potential for transformative change. Tackling these inequities requires deliberate efforts to expand African genomic representation in dental research, integrate indigenous knowledge, and strengthen sustainable local research capacity. This vision requires African-led collaborations, investment in local bioinformatics, and ethical frameworks rooted in principles such as Ubuntu. Coordinated genomic efforts, multi-site GWAS, harmonised phenotyping, and integration of microbial and environmental factors, can advance equity and precision care. Ultimately, decolonising dental genomics is not about increasing the number of African studies but about transforming the structures, values, and priorities of global health research. By centring African voices and knowledge systems, the field can build a more equitable and scientifically robust oral health landscape that truly serves African communities.

## Data Availability

The original contributions presented in the study are included in the article/Supplementary Material, further inquiries can be directed to the corresponding author.
